# Challenges, Progress, and Opportunities: Proceedings of the Filovirus Medical Countermeasures Workshop

**DOI:** 10.3390/v6072673

**Published:** 2014-07-09

**Authors:** Rona Hirschberg, Lucy A. Ward, Nicole Kilgore, Rebecca Kurnat, Helen Schiltz, Mark T. Albrecht, George W. Christopher, Ed Nuzum

**Affiliations:** 1 Division of Microbiology and Infectious Diseases, National Institute of Allergy and Infectious Diseases, National Institutes of Health, Bethesda, MD 20892, USA; E-Mails: Rona.Hirschberg@verizon.net (R.H.); hschiltz@niaid.nih.gov (H.S.); enuzum@niaid.nih.gov (E.N.); 2 Medical Countermeasure Systems, Department of Defense, Ft. Detrick, Frederick, MD 21702, USA; E-Mails: nicole.r.kilgore.civ@mail.mil (N.K.); rebecca.kurnat.civ@mail.mil (R.K.); 3 Biodefense Advanced Research and Development Authority, Assistant Secretary for Preparedness and Response, Department of Health and Human Services, Washington, DC 20201, USA; E-Mail: mark.albrecht@hhs.gov; 4 Medical Countermeasure Systems, Department of Defense, Fort Belvoir, VA 22060, USA; E-Mail: george.w.christopher.civ@mail.mil

**Keywords:** Ebola, Sudan, ebolavirus, Marburg virus, marburgvirus, *Filoviridae*, filovirus medical countermeasures, Filovirus Animal Non-clinical Group, FANG

## Abstract

On August 22–23, 2013, agencies within the United States Department of Defense (DoD) and the Department of Health and Human Services (HHS) sponsored the Filovirus Medical Countermeasures (MCMs) Workshop as an extension of the activities of the Filovirus Animal Non-clinical Group (FANG). The FANG is a federally-recognized multi-Agency group established in 2011 to coordinate and facilitate U.S. government (USG) efforts to develop filovirus MCMs. The workshop brought together government, academic and industry experts to consider the needs for filovirus MCMs and evaluate the status of the product development pipeline. This report summarizes speaker presentations and highlights progress and challenges remaining in the field.

## 1. Introduction

On August 22–23, 2013, DoD’s Joint Project Management Office for Medical Countermeasures—Joint Program Executive Office for Chemical and Biological Defense (JPM-MCS) and the HHS’s National Institutes of Health, National Institute of Allergy and Infectious Diseases (NIH/NIAID), the U.S. Food and Drug Administration (FDA), the Biodefense Advanced Research and Development Agency (BARDA), and the Assistant Secretary for Preparedness and Response (ASPR), sponsored a Filovirus Medical Countermeasures (MCM) Workshop. This 2013 Workshop was an extension of FANG driven efforts [1] and follows a 2010 government workshop focused on identifying gaps in filovirus medical countermeasure (MCM) development. The 2013 workshop brought together government scientists, policy makers, researchers, and product development experts to consider the needs for filovirus MCMs and to evaluate the status of the product development pipeline. Approximately 125 people attended the workshop, which was held in Rockville, MD, and over 200 people viewed the live videocast. A videocast recording of the workshop may be accessed online [2,3]. The workshop agenda and abstracts of the poster presentations are included as supplementary materials to this paper. 

## 2. Executive Summary

After welcoming remarks by the workshop co-chairs (N.K. and E.N.), the FANG Sub-groups presented updates on recent accomplishments, current and near-term status of projects, and lessons learned. The Assays Sub-group identified the standard Vero E6 cell line for assay development and viral propagation; and standardized the filovirus plaque assay [4]. Current activities include standardization of a quantitative polymerase chain reaction (PCR) assay, development of selected immunoassays and generation of essential assay reagents. The Well-Characterized Challenge Materials Sub-group developed rational criteria for selecting virus variants and established standardized parameters for their growth, characterization and release for use [5]. The Animal Models Sub-group continues to review ongoing animal studies on exposure doses and routes of administration, and will develop recommendations to support MCM testing and evaluation. The Animal Models Sub-group also discussed standardization of data collection, reporting parameters, and study report templates. 

The session on the conduct of adequate and well-controlled high containment or Biosafety level-4 (BSL-4) studies to support MCM development began with a presentation by the U.S. Food and Drug Administration (FDA) about the Agency’s expectations of data quality and integrity to enable regulatory approval under the Animal Rule [6,7,8]. FDA recommends compliance with good laboratory practice regulations (GLP) to the extent possible in high containment for adequate and well‑controlled BSL4 efficacy studies and for pharmacokinetic (PK) and pharmacodynamic (PD) animal studies. The FDA further recommends the use of GLP for model-defining natural history studies in support of animal model qualification. The session continued with comments from three panelists experienced in conducting BSL-4 animal studies. The panelists noted that implementing a GLP program in an academic setting requires enormous resources and committed teams; conducting GLP and non-GLP studies concurrently, particularly in small facilities, is very difficult; and equipment maintenance, validation and documentation requires on-going attention. The keys to success in conducting GLP in a BSL4 are communication, training, and collaboration with partners such as the local Institutional Animal Care and Use Committee (IACUC). The novel use of newer technologies may reduce costs and alleviate resource constraints.

The next session addressed the USG requirements for filovirus MCMs. The DoD requirements are driven by the needs of the Warfighter. The DoD seeks a trivalent filovirus vaccine that is effective against aerosol exposure and protective against filovirus disease for at least one year. Selection of a single vaccine candidate for advanced development through to FDA licensure is planned in 2017. The DoD also seeks therapeutics, enabling technologies, animal models for MCM testing, as well as threat‑agnostic treatment modalities for novel agents. The DoD is currently sponsoring advanced development of two filovirus therapeutics with planned completion of Phase 1 studies in 2014. The HHS requirements are determined by the Integrated Product Teams (IPT) established by the Public Health Emergency Medical Countermeasures Enterprise (PHEMCE), a federal coordinating body. Filovirus-specific MCM product attributes are currently being deliberated by the Viral Hemorrhagic Fever (VHF) IPT.

The session on vaccines highlighted some of the more advanced candidates from USAMRIID, Crucell Holland BV/Janssen Pharmaceuticals, Okairos/GlaxoSmithKline, Profectus BioSciences, University of Texas at Austin, the University of Hawaii, and NewLink Genetics. All of the vaccines used filovirus glycoprotein (GP_1,2_) alone or in combination with other viral proteins as antigens. All speakers presented vaccine efficacy data from non-human primates (NHP). Most groups were initiating or conducting advanced pre-clinical studies and manufacturing, although none had completed Phase 1 studies. 

The session on therapeutics focused on small molecules, monoclonal antibodies (mAbs) and gene directed therapy (siRNA and anti-sense), and highlighted the challenges of developing products through the Animal Rule [6,7,8]. Presentations were given by representatives from Tekmira Pharmaceuticals Corp., Sarepta Therapeutics, USAMRIID, Mapp Biopharmaceutical, Inc., Public Health Agency of Canada (PHAC), and University of Illinois-Chicago. The projects vary in stage of development, ranging from compound screening to proof-of-concept animal studies to Phase 1 trials. Several investigators presented promising data showing benefit when post-exposure prophylaxis was delayed one to four days after exposure, and therapeutic benefit after the onset of fever and viremia. 

The human disease session included an update from the FANG Human Sub-group, which is conducting a comprehensive literature review to better understand filovirus disease in humans, followed by presentations providing a realistic perspective on the challenges of conducting epidemiological and clinical studies in remote, resource-constrained settings. Confounding issues include imprecise case definitions, cultural factors, poor access to clinical data and care, and poor coordination among the many participating organizations. The conduct of sophisticated, well-controlled studies during outbreaks will require better infrastructure and a well-coordinated unity of effort.

The workshop concluded with a panel discussion that addressed lessons learned; gaps, challenges and bottlenecks; and perspectives on how to better facilitate filovirus MCM development. Issues highlighted included: need for a better “well-characterized” NHP model; lack of identified correlates of protection; availability of standardized assays; manufacturing concerns including process development and scale-up; importance of constructive interactions with the FDA; and challenges of product development under BSL4 containment. While there has been much progress in the past decade, many issues still face the product developer which will require determination and coordinated efforts to move filovirus MCMs forward. 

## 3. Summary of Workshop

### 3.1. Opening and Workshop Overview

Ed Nuzum (NIH/NIAID), FANG and workshop co-chair, welcomed participants and outlined goals of the workshop. He noted the emphasis was on products, both vaccines and therapeutics, and that the field is immature but progressing. The intent of the workshop was to highlight progress made in product development and identify the gaps or challenges to the product developer.

Nicole Kilgore (JPM-MCS), FANG and workshop co-chair, described the structure and goals of the FANG. The FANG is a part of the Portfolio Advisory Committee, a higher level interagency group that coordinates and oversees USG MCM portfolios. The FANG is a diverse group, with the mission of developing consensus recommendations for the standardization of reagents, methods and procedures across multiple agencies and laboratories, and to develop strategies to address broadly applicable and interagency product development issues relevant to licensure of filovirus MCM [1]. The FANG is not a policy group but one that produces data and facilitates research and development of both vaccines and therapeutics. One mechanism for achieving these goals is through FANG-supported workshops. 

### 3.2. FANG Sub-Group Updates; Rebecca Kurnat, Chair

Rebecca Kurnat (JPM-MCS) represented the FANG Assays Sub-group, which includes participants from DoD, HHS, FDA, Texas Biomedical Research Institute (TBRI), University of Texas Medical Branch (UTMB), Public Health England, and Defence Science and Technology Laboratory. The focus of Assays Sub-group is standardization of assays and reagents using a collaborative, iterative approach that involves collecting and analyzing available protocols, developing consensus protocols, and the testing, refining, and transferring of assays to participating laboratories. This approach has led to a standardized plaque assay for filoviruses [4] and a quantitative PCR assay. Immunoassays are under development and large batches of essential assay reagents are being prepared. A standard VeroE6 cell line for assays is also available from DMID’s Biodefense and Emerging Infections Research Resources Repository (BEI Resources, NR-596, lot 3956953). 

Lucy Ward (NIH/NIAID), representing the FANG Well-Characterized Challenge Materials (WCCM) Sub-group, discussed the group’s efforts to identify, characterize and produce exposure materials appropriate for use in MCM product development. The selection of filovirus virus variants representative of the ebolaviruses and marburgviruses was achieved through the development of a group consensus list of desired viral stock characteristics/traits which was subsequently subjected to an algorithmic analysis to assign weights to each listed criterion/trait. The top three weighted criterion for selection of founder virus stocks (to master and working viral banks for MCM testing studies) were: the isolate must come from a lethal case and preferably from an outbreak involving multiple persons; the isolate must be of low passage from the human case and not have been passaged in animals; and the material must be well-characterized and of known wild-type genomic sequence [9]. First passage aliquots of Ebola virus (EBOV) variant Kikwit, Sudan virus (SUDV) variant Gulu and Marburg virus (MARV) variant Angola isolates were then obtained from the Centers for Disease Control and Prevention (CDC) and draft methods to grow these viruses established. For current (near-term) MCM development and other uses, P2/P3 (second and third passage) master stocks/working stocks of these virus isolates have been made available to BSL-4 facilities. Characterization and release criteria for the master and working banks were developed and include potency evaluation by the standardized plaque assay, identity confirmation by PCR, and sterility, mycoplasma, and endotoxin testing. Viral batches will be deep sequenced following WCCM recommended guidelines and standards [5], tested for infectivity and lethality in animals, and evaluated for stability. The WCCM’s group focus continues on further delineation and definition of virus characterization methods and traits of these human clinical isolates with efforts initiated to investigate standardization of small animal-adapted exposure materials. 

William Dowling (NIH/NIAID) discussed the FANG Animal Models Sub-group’s progress in facilitating development of well-characterized animal models for MCM testing. Topics of interest include defining appropriate exposure doses and routes for the indication; identification and establishment of models that recapitulate human disease; determination and standardization of trigger to treat and euthanasia endpoints; standard assays; and standardized data collection. More work is needed to define exposure doses, particularly to substantiate the historically used 1000 pfu dose. Although LD_90_ and LD_99_ studies are likely feasible, LD_50_ studies may not be feasible due to technical issues and costs. Dosing studies are in progress using different animal species. Standards for data collection and reporting are critical; a reporting template and a template for capturing individual animal case report forms are nearing completion. Regarding euthanasia criteria, the group discussed the use of different criteria by each site and the impact of this practice on outcomes. Consistent use of defined euthanasia criteria at each facility must be implemented and increased monitoring of animals in the critical period by all groups is needed and will be discussed further. While individual IACUCs and an institution’s attending veterinarian have final authority, recommendations by the community would carry weight and facilitate harmonization of practices. A workshop was held in early 2014 to further discuss the group’s topics of interest [10]. A comment from the audience suggested that the ability to predict efficacy of a MCM in humans is an important consideration in animal testing. This is a critical issue, especially for host receptor-directed therapeutics, because of varying degrees of conservation among analogous receptors and host targets. 

### 3.3. Conduct of Adequate and Well-Controlled BSL-4 Studies during MCM Development; Lisa Hensley, NIH/NIAID, Chair

This session focused on the challenges associated with conducting studies in BSL-4 containment that will satisfy regulatory requirements.

Andrea Powell (FDA/CDER/OCTEC) talked about FDA’s expectations of data quality and integrity for Animal Rule-specific studies. Confusion exists about FDA’s expectations for GLP with these and other related studies based on earlier documents (see 67 *Federal Register* 37988 at 37989, May 31, 2002 and FDA’s 2009 draft guidance for industry *Animal Models—Essential Elements to Address Efficacy Under the Animal Rule*) [6,7]. FDA currently recommends the use of GLP, to the extent practical, for the adequate and well-controlled animal efficacy studies and the PK and/or PD studies in animals used to select a dose and regimen in humans. FDA also recommends the use of GLP for the model-defining natural history studies that are submitted to support the qualification of an animal model through FDA’s Animal Model Qualification Program. Assurance of data quality and integrity for these three types of studies is critical since they serve as the basis for regulatory decisions. FDA acknowledges that there may be justifiable limitations in the ability to apply GLP when conducting these studies, especially those requiring use of maximum containment facilities. Before initiating these studies, sponsors should identify aspects of the studies anticipated to be a challenge with regard to GLP, propose methods for adapting the studies to ensure the quality and integrity of the resulting data, and seek concurrence from FDA on the plan. Inspections will be conducted to verify quality and integrity of the raw data, the supporting documentation, and the results submitted in the final study report. FDA will verify that study personnel followed the agreed upon data quality and integrity plan such that each phase of the study can be reconstructed. Sponsors should contact the appropriate review division within CBER or CDER for specific advice about studies for investigational products, and contact the Animal Model Qualification Program for advice about the qualification of product‑independent animal models. In response to a question about whether “GLP-like” studies could be acceptable to FDA, Dr. Powell said that it depends on what the differences from GLP are and how those differences would affect the data. This information should be provided to the review division. The decision as to whether a study is acceptable is the purview of the review division. Again, it is best to have discussions with the review division regarding this issue prior to the initiation of the study. 

Three panelists with experience in conducting BSL-4 studies that are GLP compliant or GLP-like, discussed their experiences and lessons learned. 

Louise Pitt discussed USAMRIID's efforts to develop GLP capability at BSL-4. These efforts were begun several years ago and built upon having had GLP at BSL-3 for 10 years. As a result of equipment needs and the importance of backup, all three BSL-4 suites are GLP-compliant. The first “mock-GLP”studies (GLP but without quality assurance (QA)) were done last year. A GLP BSL-4 animal study that included both aerosol and intramuscular routes of exposure has been done. Several other product-independent studies are pending. In several years USAMRIID will be moving into new facilities that will integrate GLP capabilities such as electronic data handling. USAMRIID researchers have learned that GLP requires an enormous and sustained commitment. It is very difficult to conduct both GLP and non-GLP research concurrently in the same facility. Consequently, USAMRIID plans to conduct all research using “mock-GLP” conditions. Issues relating to equipment and documentation were the biggest hurdles. Having backup equipment to minimize lost time when there are failures is crucial. Communications are essential, and continuous training for scientists to maintain compliance must be in place. 

Ricardo Carrion discussed the challenges at the Texas Biomedical Research Institute (TBRI) which paralleled many of those already mentioned. Further, because TBRI is a small facility, resource issues are particularly pressing. They can now do well-characterized and well-controlled NHP studies to support development under the Animal Rule, but they have not yet performed a GLP-compliant animal study in the BSL-4. They are focusing on technology to reduce space and personnel constraints, such as the use of closed circuit TV for quality control (QC). Re-education of staff is a continuous effort that requires the engagement of a dedicated QC-QA staff. Efforts to harmonize quality assurance agreements with several sponsors are on-going. 

Trevor Brasel reviewed UTMB’s experience. They have completed their first, adequately-controlled, well-documented study using NHPs in BSL-4. Though it was not GLP, the principles of GLP, particularly the use of standard procedures, protocols, and documentation/QC practices, were implemented. Bringing GLP practices into an academic environment is challenging, and building a foundation for GLP compliant studies requires an enormous effort. There is a knowledge gap between the contract research organization (CRO) and the academic communities. Training and outreach about scientific research *versus* applied regulated work, proper documentation practices, the development of standard operating procedures (SOPs) and standard documentation practices are ongoing. These efforts include the use of standardized forms and workbooks. Quality control of data is challenging to coordinate and implement. The reality that GLP-compliant studies may require additional time and effort compared to basic research may be difficult to accept. Educating the IACUC about the conduct of GLP studies in high containment is important, particularly when NHPs are involved. UTMB sponsored a workshop and an FDA-sponsored course in April 2013. Another course will be offered in 2014 in the Washington, D.C. area. 

Additional points were made during the discussion: GLP-compliant studies require multidisciplinary teams, including specialized quality teams and core units. GLP must be an institute‑wide and continuous commitment. One approach is to cross-train researchers to do QC as an additional duty. Inventory control and tracking should begin when equipment items are first received. QC and required documentation can add at least 40%–50% to study costs. Telemetry can reduce, but not eliminate, the need to enter the BSL-4 laboratory. Working with the IACUC can help resolve monitoring issues. Data transfer out of laboratories can be enhanced by several approaches including the use of data sheets that can withstand chemical decontamination or irradiation, and the use of electronic records. Having inspectors in the BSL-4 laboratory is problematic due to occupational health considerations including the risk of inadvertent exposures and the use of positive-pressure suits. Windows and closed-circuit television enable inspectors to make limited observations, but may not be enough. 

Dave Parish from NIH/NIAID’s Integrated Research Facility at Fort Detrick talked briefly about information technology-related issues and GLP. They have developed an integrated system of programs to plan and track every aspect of research from protocol development to electronic data transfer. This system facilitates GLP compliance, and also results in better quality data that are useful to researchers for a longer period. 

### 3.4. Government Agency Requirements for MCM; Nicole Kilgore, Chair

This session focused on DoD and HHS requirements for filovirus MCM and the government procedures and processes involved in MCM development.

Nicole Kilgore (JPM-MCS) described how the DoD generates requirements and provided an overview on the DoD filovirus vaccine efforts. The process is driven by warfighter needs and their requirements developed from a general need concept and then moves into specifics. Requirements must balance wishes with reality. Most projects involve licensure under the Animal Rule pathway. The current draft vaccine requirement is for a single vial, pre-exposure trivalent vaccine that will protect against a 1000 pfu filovirus exposure by aerosol. Efficacy for post-exposure prophylaxis (PEP) would be desirable. Immunity should last at least one year, but preferably five, and a shelf-life of two years is needed, with five being preferred. MCS-Joint Vaccine Acquisition Program (JVAP) is supporting the development of two vaccine prototypes, the Venezulan equine encephalitis virus (VEEV) replicon particle (VRP) and a non-replicating virion like particle (VLP). At the end of Phase 1 clinical studies (also known as Milestone B), projected to conclude in 2017, the MCS-JVAP will make a down‑selection among these and other government or industry candidates. DoD efforts are being coordinated with HHS filovirus vaccine activities.

George Christopher (JPM-MCS) discussed DoD’s requirements for filovirus therapeutics, technologies, and animal models to respond to novel agents. Pre-clinical therapeutic candidates have included threat-agnostic modalities such as host-based therapies to interrupt viral life cycles or common pathways of pathogenesis. Two pathogen-directed therapeutic candidates in advanced development are based upon antisense and siRNA backbones that can be rapidly adapted to target newly identified genetic sequences. In addition to providing specific therapeutics, these projects provide proof-of-concept for tools that enable rapid rational drug design. Milestone B for these efforts will be in 2014. The goal is regulatory approval of a filovirus therapeutic product in 2018. MCS also supports animal model development to test vaccines and therapeutics, and to complement animal model efforts sponsored by HHS. 

Christopher Van De Wetering (HHS/ASPR) discussed the government’s civilian requirements for filovirus MCMs. This effort is facilitated by the Public Health Emergency Medical Countermeasures Enterprise (PHEMCE). The PHEMCE is a federal coordinating body led by HHS that works to optimize preparedness for public health emergencies through the creation, stockpiling and use of MCMs. Civilian MCM requirement setting is coordinated by the Division of Medical Countermeasures Strategy and Requirements (MCSR) within HHS/APSR via nine threat-specific integrated program teams (IPTs) including the relatively new VHF IPT. The PHEMCE requirements process begins with a Department of Homeland Security-led assessment of the threat that includes the number of individuals likely to be exposed to a threat agent in a series of plausible, high-consequence scenarios; this information is used to determine the magnitude of adverse health impacts resulting from the release of a threat agent. Subsequently, medical consequence modeling is used to identify the requisite amount of MCM needed to mitigate the adverse health consequences identified in the scenario. In concert with the formulation of requirements, the establishment of MCM product attributes, utilization plans, and identification of capabilities, gaps and solutions are undertaken. The 2012 PHEMCE Strategy and Implementation Plan discusses these processes in greater detail. Filoviruses have been identified as a material threat to national health security. The PHEMCE has established filovirus-specific MCM requirements, and the VHF IPT is currently elaborating on MCM product-specific attributes.

### 3.5. Vaccine MCMs: Presentations by Sponsors of Vaccine Candidates; Lucy Ward and Pat Repik (NIH/NIAID), Chairs

This session highlighted the status of filovirus vaccine efforts with presentations by investigators who are working on some of the most advanced products currently in the pipeline.

Sina Bavari (DoD-USAMRIID) described his team’s work on a VLP vaccine for filoviruses. VLPs are virus-sized particles formed by viral proteins (lack nucleic acid) and thus retain virus morphology but are noninfectious. VLPs can be produced in large quantities and generate robust innate, humoral and cellular immunity in rodents, NHPs and humans. A VLP vaccine to prevent diseases due to human papilloma viruses has been licensed by FDA. An advantage of VLP vaccines is that there are no issues regarding vector immunity. The Bavari team has developed protocols to make large quantities of well‑characterized material; the key antigen is filovirus GP_1,2_. VLPs with EBOV or MARV antigens enter dendritic cells (DCs) and induce maturation. VLPs activate NK cells and induce DCs to produce protective immunity; this process involves NKp30. VLPs induce protection against EBOV in mice and guinea pigs as determined by survival following a 1000 pfu exposure [11]. VLPs provided post‑exposure protection to NHPs when given up to 3 days after infection. MARV VLP vaccinations protect mice, guinea pigs and NHPs [12]. This protection results in long lasting immunity and is independent of perforin, but dependent on CD4^+^ and CD8^+^ T cells. Interference between EBOV and MARV VLP vaccines has not been observed when given either simultaneously or sequentially. Vaccination using MARV VLPs and two different adjuvants provided protection in NHPs against MARV with a 1000 pfu aerosol exposure. The Bavari team produces stable VLPs of uniform size that can be purified by filtration. The amount of GP_1,2_ in these VLPs can be quantified by ELISA and mass spectrometry. Dosing studies over a range of 300 to 0.1 µg showed protection down to 1 µg. Preparations for good manufacturing practices (GMP) production and GLP toxicity studies in 2015 and, possibly Milestone B in 2016 are underway. Their goal is to produce a single vaccine effective against EBOV, SUDV, and MARV. In response to a question, Dr. Bavari said that protection in animals lasts at least 6 months. 

Gene Olinger (NIH/NAID, formerly DoD-USAMRIID) discussed his team’s work on VRP vaccines while at USAMRIID and supported by funds from the NIH, DoD’s Joint Science and Technology Office, and the former DoD Chemical and Biological Medical Systems (now MCS). A helper-cell system was used to make VEEV expressing filovirus GP_1,2_. The platform has been tested using other antigens in humans. Early studies showed that GP_1,2_ is a protective antigen. Studies to characterize the immune response to filovirus-VEEV vaccines identified mAbs that can be used for passive immunotherapy. An effective pan-ebolavirus vaccine will need multiple GP_1,2_ components, because the analogous proteins differ among the five ebolaviruses and do not confer cross-protection against heterotypic viruses. Marburgvirus GP_1,2_s are more highly conserved, and induce cross‑protection. Proof of concept for a VEEV VRP filovirus vaccine was demonstrated in 1998 with MARV in NHPs. Immunization of guinea pigs with 10^8^ infectious units of MARV variant Ci67 resulted in survival. Similar results were obtained after challenging NHPs by intramuscular (IM), subcutaneous (SC) or aerosol challenges 28 days after vaccination, although some animals developed clinical signs. Up to 50% survival was observed when vaccination was given post-exposure. Past efforts to advance an EBOV product were confounded by dosing problems and unclear results. However, vaccine doses greater than 10^8^ VRPs were effective in NHPs. VRPs with GP_1,2_ antigens from variants of EBOV and SUDV conferred survival among cynomolgus macaques [13]. Immunogenicity and efficacy have been shown using a pan filovirus product. One dose of 10^10^ VRPs or multiple doses of 10^8^ VRPs are effective. Efficacy has also been demonstrated in MARV aerosol exposure studies following vaccination with 10^9^ VRPs expressing a MARV GP_1,2_. Cell-mediated immunity is important in mice, and likely guinea pigs and NHPs. Protocols for GMP production are established and production anticipated in 2014; Phase 1 studies are projected in 2015. Work to define correlates of protection, to develop assays for pre-clinical and clinical studies, to define vaccine dosage and schedule, as well as bridging studies are planned. A portfolio of approaches that includes vaccines, post-exposure prophylaxis (PEP), diagnostics, and therapeutics is desirable to address diseases caused by filoviruses. In response to questions, Dr. Olinger mentioned that the possibility of a conserved ebolavirus GP_1,2_ antigen should be investigated. Issues regarding effective vaccine doses and the economic costs of manufacturing VRPs need to be addressed. 

Benoit Callendret (Crucell Holland BV/Janssen Pharmaceuticals) described the company’s work on advanced development of a multivalent filovirus vaccine based on recombinant adenovirus (Ad) vectors Ad26 and Ad35 that infect humans at low seroprevalence. Studies using recombinant Ad vectors expressing antigens from other human pathogens such as HIV-1 [14,15,16], *M. tuberculosis* and plasmodia demonstrated good safety profiles in large groups of people with doses up to 10^11^ vector particles (vp). GMP vaccine can be manufactured using a low volume, high density “process intensified” process. Current efforts are at the 50-l scale, with the goal of reaching a 500-l scale. Phase 1 studies of a trivalent vaccine are planned for the end of 2016. Protective efficacy studies to date have all involved an Ad26 prime and an Ad35 boost with various viral GP_1,2_ antigens, followed by exposure four weeks after the boost immunization [17]. A study in cynomolgus macaques vaccinated with a quadrivalent vaccine composed of GP_1,2_ from EBOV, SUDV, MARV, and Taï Forest virus (TAFV), followed by IM inoculation with MARV variant Angola demonstrated 100% survival with a high-dose vaccination regimen (10^11^ vp each) and 75% survival with a low dose regimen (2 × 10^10^ vp each). GP_1,2_ ELISA titers correlated with survival. Data from EBOV variant Kikwit exposures are less clear. Historical data from vaccine efficacy studies performed at the USAMRIID BSL4 facility using a bivalent antigen vaccine composed of EBOV GP_1,2_ and SUDV GP_1,2_ (10^11^ vp each) fully protected NHPs from death following an intramuscular exposure dose of 1000 pfu of the USAMRIID EBOV variant Kikwit virus material but in a follow-up study at the TBRI BSL4 facility using the same vaccination regimen and a 1000 pfu exposure dose but of wild-type EBOV variant Kikwit material, only 33% of the NHPs survived. Such discrepant outcomes highlight the need for well-characterized exposure material in order to replicate and compare vaccine efficacy studies across BSL4 facilities. A study with tetravalent or trivalent vaccines using doses of 3 × 10^10^ or 4 × 10^10^ vp for each antigen demonstrated 50% survival after EBOV exposure. ELISA titers correlated with survival. Optimization of the EBOV vaccination regimen is in progress. A trivalent vaccination regimen at 4 × 10^10^ vp for each antigen conferred 75% survival after exposure to SUDV variant Gulu exposure. Overall, the studies showed better protection for SUDV and MARV than for EBOV. In response to a question, Dr. Callendret said that all studies were either completely or partly blinded. He confirmed that the cell line used for vaccine production is qualified for GMP manufacturing.

Alfredo Nicosia (Okairos/GSK) described studies with filovirus vaccines based on replication-incompetent chimpanzee adenovirus (ChAd) and modified vaccinia Ankara (MVA) vector technology. This approach uses ChAd vectors to obviate the issue of background immunity to human Ad5 vectors [18]. A large group of ChAds was isolated, and ChAd strains with immunological potency equivalent to Ad5 were identified and further developed. Heterologous prime/boost regimens with ChAd and MVA showed improved immunological response over ChAd priming alone in NHPs and in human clinical trials of vaccine candidates to prevent malaria [19] and HCV and HIV-1 infections. Both ChAd and ChAd/MVA regimens appear to be safe in humans. Overall, there seems to be a good correlation between immunogenicity in NHPs and humans. A single dose of 10^10^ vp of ChAd3 encoding the EBOV GP_1,2_ induced T and B cell immune responses in NHPs similar to those seen with the human Ad5 vector, and fully protected NHPs exposed four weeks post-vaccination to an intramuscular injection of 1000 pfu of a USAMRIID EBOV variant kikwit. Similarly, doses of 10^10^ or 10^9^ vp of the corresponding ChAd3 encoding MARV GP_1,2_ conferred protective immunity to NHPs. The NHPs’ GP_1,2_ antibody titers correlated with survival, and no interference was observed in co‑administration studies with two ChAd3 vectors encoding different GP_1,2_. Exposure (intramuscular route) of vaccinated NHPs to 1000 pfu of the USAMRIID EBOV variant kikwit one year post‑vaccination resulted in 50% survival with a single administration of 10^11^ vp ChAd3-GP, and use of a ChAd3-GP prime with an MVA-GP boost regimen conferred 100% survival of NHP after 1 year. Pilot scale manufacturing processes are in place, and GMP lots of vectored vaccines have been made for planned clinical studies. There were questions from the audience on the need for long term protection, and on how vaccine vectors derived from chimpanzees might be perceived. The safety profile of ChAd vectors in European clinical trials involving more than 1000 humans to date is promising. Finally, the need for standardization of the filovirus NHP exposure model at different BSL4 sites was emphasized. 

John H. Eldridge (Profectus BioSciences) presented his team’s recombinant vesicular stomatitis Indiana virus (rVSV) vectored vaccines for EBOV and MARV. Their highly attenuated, genetically modified rVSV vector is a replicating virus with good immunogenicity and low virulence. It is suitable for commercial scale manufacturing, and several clinical trials using this vector with other antigens have demonstrated safety. Repeated doses of homotypic rVSV resulted in boosted antibody titers. Several variants of the vector have been developed, to enable the sequential use of multiple rRSV-based vaccines using heterologous vectors. This strategy may mitigate the risk of poor of immunogenicity in vaccine recipients with immunologic memory to vector variants delivered in previous vaccinations. The VSVN4CT1 vector strain was used in the presented studies. Neutralizing antibody titers can be obtained after one 10^6^ vp dose. A pan-filovirus vaccine can be delivered in one dose. Historical studies done with a rVSV vector-GP_1,2_ construct (delta G) conferred 100% survival after 1000 pfu exposures to MARV or EBOV. In addition, the rVSV candidates conferred survival benefit when given as post-exposure prophylaxis [20,21,22]. They compared the delta G vector with the new rVSV with EBOV GP_1,2_ at a 10^7^ dose in a guinea pig challenge study and conferred complete survival using both vectors. There was 100% survival in a NHP study, and 66% cross-protection against SUDV. These studies were done with the FANG designated isolates of EBOV variant Kikwit and SUDV variant Gulu as exposure materials. When the rVSV-GP construct was engineered to produce anchored protein, complete protection was observed and high Ab titers were measured However, when a secreted version of GP_1,2_ was used in the rVSV construct, incomplete protection against MARV exposure was observed and animal’s developed lower antibody levels to the vaccine. Antibody-mediated immunity seems to be more important than cell-mediated immunity. The long-term goal is to develop a trivalent vaccine suitable for the strategic national stockpile. Phase 1 studies could begin in 2016. In response to a question about longer protection, he said that the duration of protective immunity has not yet been determined. 

Maria Croyle (University of Texas at Austin) discussed her team’s evaluation of mucosal vaccination against EBOV using an Ad5-based vaccine. The goal is a vaccine that provides systemic and mucosal immunity with memory, low toxicity, and ease of administration and delivery. They have been exploring nasal and oral delivery, to obviate pre-existing immunity to Ad5. They have developed a first-generation Ad5 vector that expresses EBOV GP_1,2_. The construct is highly immunogenic, with doses as low as 10^4^ vp conferring protective immunity in mice. The current formulation is stable after five freeze-thaw cycles, after two years of storage at 4 °C, and for nine hours at room temperature. The formulation also prevents aggregation and does not dampen immune response in mice. Intranasal administration produced an immune response comparable to that induced by intramuscular dosing in mice. NHP studies have been conducted in collaboration with Dr. Kobinger’s group at the Public Health Agency of Canada. Intranasal vaccination using a dose of 10^10^ particles induced both cell-mediated and humoral responses. Shedding of the vector was limited to the immediate post-vaccination period. Survival among vaccinated NHPs was 100% at 28 days, and over 60% at 62 days post-challenge. These data, with other data generated by Dr. Kobinger’s team [23] have facilitated the launch of a clinical trial that is planned for 2014 in Canada. With respect to pre‑existing immunity to Ad5, mice that were immunized with Ad5 prior to filovirus vaccination demonstrated impaired CD8^+^ responses. However, the route of dosing made a significant difference; homotypic routes for the induction of immunity to Ad5 and subsequent immunization with the Ad‑5-EBOV construct resulted in lower immune responses and survival than when different routes were used. The team’s nasal formulation performed well in mice with pre-existing immunity, and gave 80% survival [24]. Virus-specific titers of IgG1 seem to be particularly important for survival. An oral vaccine candidate (for buccal or sublingual administration) conferred 80% survival among naïve mice, and 100% among those with pre-existing immunity [25]. This finding was unexplained, but will be investigated further. 

Axel Lehrer (University of Hawaii) discussed studies employing recombinant filovirus antigens as vaccines. The program was started at Hawaii Biotech, Inc., continued development at Panthera Biopharma, LLC, and now is being pursued at the University of Hawaii. Advantages of the subunit approach include the ability to precisely select antigen doses and the elimination of translation of protein antigens in the host. The platform has previously been used for other vaccines. Subunit proteins can be formulated with various adjuvants, which may reduce requirements for mass production of antigens. His team’s approach produces antigens in a *Drosophila* expression system (stably transformed cell lines) which yields glycosylated proteins that are subsequently purified by immune‑affinity chromatography. Steps in subunit vaccine development include: antigen selection, production, immunogenicity testing, efficacy testing and ultimately formulation optimization for each host species. Filovirus proteins GP_1,2_, VP24 and VP40 with various adjuvants were tested in animals. No significant toxicity has been observed in rodents and non-human primates, and proof-of-concept for immunogenicity and efficacy has been established in several animal models [26]. In the EBOV model, mice receiving a 10 µg dose of GP_1,2_ without adjuvant showed 70% survival; when all three antigens were given this increased to 90%. With various adjuvants 100% protection was observed. While GP_1,2_ alone can confer 100% survival, VP24 also protective, most likely due to cellular immunity. Serum transfer from GP_1,2_-immunized animals as well as T-cell transfer from animals immunized with GP_1,2_ or VP24 gave significant levels of protection in mice. Guinea pig studies were used to optimize combinations of antigens and adjuvants. Experiments suggested that proper antigen balancing is required because excessive antigen reduced efficacy. Survival was correlated with antibody titers at the time of exposure. A NHP study with an emulsion-based adjuvant using the 3 antigens at a moderate dose level showed 50% survival after EBOV exposure, and survivors had a significant reduction in viremia compared to non-protected vaccinees. Data further suggested that potent anamnestic responses during exposure are important characteristics of a successful vaccination. Expression cell lines, mAbs for purifying proteins, and purification methods have been selected and developed. GMP production for clinical trials could start within 1.5 years. GLP toxicity studies would require approximately one year, and Phase 1 clinical testing could commence within 4 years.

Jay Ramsey (NewLink Genetics) discussed BPSC1001, a post-exposure and pre-exposure prophylaxis for EBOV. The platform is an rVSV with the VSV-G envelope protein gene removed (VSV-deltaG) and EBOV variant Kikwit GP_1,2_ gene inserted. This attenuates VSV replication, but the vaccine reaches titers greater than 10^8^ vp per mL. The vector was developed by reverse genetics and patented at Special Pathogens Program, Public Health Agency Canada (SPP, PHAC) with the IP being licensed to BioProtection Systems/NewLink Genetics. Pre-exposure vaccination conferred survival to rodents and NHPs after a 1000 pfu exposure; multivalent vaccines did not exhibit significant interference, although balancing may be required. Vaccine was effective given by intramuscular, oral, or intranasal routes. Correlates of protection are antibody and T cells responses. A window for post‑exposure effectiveness was demonstrated, although the mechanism was not established. In pre-clinical studies, there were no adverse events in NOD SCID immunodeficient mice or animals infected with SHIV (a human-simian immunodeficiency virus construct), and no neurotoxicity was observed in NHPs. The PHAC sponsored GMP-compliant vaccine production in 2013, and the process appears scalable up to at least 15,000 doses. Pre- and post-exposure studies in mice have been done, and NHP exposure studies are ongoing. GLP toxicity studies, IND submission, and Phase 1 are planned for in 2014. The regulatory plan is for a PEP indication with a multivalent product, starting first with BPSC1001 for ebolaviruses, multivalent products next and a pre-exposure indication at a later time. The initial Phase 1 clinical designs for safety, toxicity and immunogenicity are complete. The product has an immediate application for lab workers, but the primary customers would likely be government and humanitarian organizations desiring to stockpile products for emergency use. In response to a question it was indicated that good response durability data out to 14 months have been reported.

### 3.6. Therapeutic MCMs: Presentations by Sponsors of Therapeutic Candidates; Helen Schiltz (NIH/NIAID/DMID), Chair

This session focused on several therapeutic approaches, including small molecules, mAbs, and alternative delivery platforms, and highlighted the challenges of developing products under the Animal Rule. 

Ian MacLachlan (Tekmira Pharmaceuticals Corp.) discussed lipid nanoparticle (LNP)-based siRNA therapeutics for viral hemorrhagic fevers. Tekmira’s platform technology uses LNPs to deliver siRNAs that inhibit viral gene expression, thereby reducing viral replication. LNPs are made of amino lipids, structural lipids, phospholipids, polyethylene glycol-lipids, and nucleic acids. They are easily manufactured using an automated process to give a colloidal suspension of particles with consistent diameters of approximately 80 nm. The process is currently scaled to manufacture clinical and commercial quantities. siRNA-encapsulated LNPs enter cells through receptor-mediated endocytosis utilizing low-density lipoprotein receptors and related receptor family members. Once released into the cytoplasm, siRNAs bind to host RNA-induced silencing complexes to catalytically destroy the targeted mRNA. Tekmira’s LNP technology is also being used to develop cancer therapies, and has been validated in seven clinical trials. The ability to encapsulate multiple siRNAs in an LNP may enhance the efficacy and spectrum of filovirus therapeutic candidates. Tekmira is developing candidates for both MARV and EBOV, with an emphasis on EBOV variants. The Tekmira EBOV candidate is active *in vitro* and in guinea pig and NHP animal models. LNPs targeting VP35, VP24, and L in various combinations have been studied. An NHP study demonstrated 100% survival when treatment was initiated 1 hour post-infection and was continued using seven daily doses of 0.5 mg/kg. Treatment prevented detectable viremia, and greatly reduced histopathologic findings, and abnormalities of liver function [27]. Tekmira plans to conduct Phase 1 clinical studies using a reformulated candidate in 2014. In response to a question about exposure material, Dr. MacLachlan responded that USAMRIID EBOV variant Kikwit material is being used which may represent a cell-culture EBOV variant (8U), as opposed to a low-passaged EBOV that has undergone limited cell culture passages and genetically more similar to the clinical isolate (7U). Tekmira will follow developments in the FANG for guidance regarding exposure material to be used in future studies. In response to other questions, Dr. MacLachlan commented that the new LNP formulation shows lower toxicity and higher potency than older versions. It may be possible to encapsulate multiple siRNAs within an LNP (mixed cocktail) to develop a pan-filovirus product. It is also possible to develop LNPs that target specific cell types. Dr. MacLachlan reiterated the need to identify sensitive and specific biomarkers to determine a trigger to treat. 

Jay Charleston (Sarepta Therapeutics) discussed AVI-7288, an antiviral candidate for MARVinfection. The product is a synthetic polymorpholino oligonucleotide (PMO) compound that interrupts translation. The PMO chemical backbone is a synthetic polymer that takes the place of ribose to develop oligonucleotide-based therapeutics. Sequences of nucleoside bases can be arranged to target any gene. The current drug, AVI-7288, is a PMO*plus*^™^ with five positive charges (to enhance intracellular delivery) that targets the filoviral nucleoprotein [28]. Treatment consisting of 15 mg/kg/day of drug given intravenously (IV) 1 hour after a 1000 pfu MARV variant Musoke exposure conferred 83% to 100% survival among cynomolgus macaques, compared to a uniformly lethal outcome among control animals. Delayed treatment at 15 mg/kg/day given IV at 24, 48, or 96 hours after exposure for 14 days and intramuscular dosing at 30, 15 and 7.5 mg/kg/day starting 1 hour after viral exposure all gave significant protection [29]. Control animals were also included in natural history studies to validate the disease model as required under the Animal Rule. A GLP study performed under BSL-4 containment showed the PK profile of AVI-7288 was comparable across doses in healthy and infected animals. This PK profile will support the determination of effective human doses. PK profiling of intramuscular dosing suggests that total exposure may be more predictive of efficacy than maximum concentrations. Genetic sequencing of viruses isolated after drug exposure indicated no selective pressure for the development of resistance. A single ascending dose Phase 1 clinical study in healthy human volunteers has been completed and indicates that the drug is well tolerated and safe at doses administered. A multiple ascending dose clinical trial is in progress and will guide the determination of a safe and effective dose ranges in humans.

Travis Warren (DoD-USAMRIID) discussed the development of small molecule nucleoside analogs to inhibit viral replication. Advantages of small molecules include lower costs of goods, ease of delivery, favorable tissue distribution, and amenability to structure activity relationship (SAR) studies that may enable to compound optimization and rational drug design. Although many nucleoside analog drugs have been FDA-approved to treat other viral diseases, the search for a filovirus small molecule therapeutic is ongoing. Dr. Warren and his team are studying the nucleoside analogue BCX4430, which has broad-spectrum antiviral activity [30]. The lead compound has favorable drug-like properties and a known mechanism of action. It is not incorporated into mammalian RNA or DNA, and is not mutagenic in Ames tests. Proof-of-concept studies in yellow fever disease models demonstrated efficacy when given up to four days after exposure. Activity against multiple filoviruses has been demonstrated in NHP PEP studies. Pre-clinical IND-enabling studies have started.

Larry Zeitlin (Mapp Biopharmaceutical, Inc.) discussed a mAb cocktail (MB-003) for use against EBOV. Numerous mAb-based products have been FDA-approved and are widely marketed for use in oncology, gastroenterology, rheumatology and clinical immunology, as well as infectious diseases; consequently, costs are dropping. Mapp has nine mAb projects in progress. The three components of MB-003 are humanized, murid-derived antibodies that target non-overlapping regions of EBOV GP_1,2_. One mAb is cross-reactive with the GP_1,2_ of SUDV, Reston virus (RESTV), and TAFV. The mAbs are manufactured in plants via a Rapid Antibody Manufacturing Process, which is both faster and less expensive than using mammalian cells. Approximately 1 kg of GMP-grade mAb can be produced per month. The plant-produced antibodies are more potent in murine and NHP studies than mammalian-cell expressed mAbs. This may be due to differences in glycosylation in the two expression systems. The mechanism of action utilizes antibody-dependent cell-mediated cytotoxicity, and is independent of complement activation. The cocktail is protective in mice both pre- and post-infection (2 days). NHP studies demonstrated efficacy with 50 mg/kg total antibody given at 1, 24 or 48 hours after exposure [31]. Significant benefit was achieved even when treatment was delayed until the appearance of fever and viremia [32]. The antibody half-life in animals is about 7.4 days. PK studies suggest that the mAbs are consumed as viral load increases, opening the possibility of further dose optimization. Non-GLP pre-clinical safety studies are being done, and other pre-clinical work is planned for 2014. MappBio is starting a joint venture with Defyrus for further development. With sufficient funding, an IND submission could be filed in early in 2015. 

Gary Kobinger (Public Health Agency of Canada) discussed therapeutic mAbs against EBOV [33]. A critical aspect of developing such treatments is the need to identify a window of opportunity to treat before viral load and clinical severity are overwhelming. In mice, guinea pigs and NHPs there is a high correlation between antibody titers and survival, but both humoral and cell-medicated immune responses are desirable. Their product contains three murine mAbs specific for GP_1,2_ with EBOV-neutralizing activity, and are not cross-reactive with other ebolaviruses, such as SUDV. Immunologic escape mutants have been detected in approximately one animal per 50 treated nonhuman primates. The issue of exposure material is critical; they are using 1000 pfu EBOV variant Kikwit that is predominantly 7U (wild type). A NHP study using cynomolgus macaques, demonstrated 100% survival if the first mAb dose was given at 24 hours, and 50% with dosing started at 48 hours. Most surviving animals survived a re-exposure 10 weeks later; survival correlated with convalescent antibody titers [33]. The half-life of the mAb combination ranged between 38 and 53 hours. The Kobinger team is also investigating DEF201, a broad spectrum antiviral which might be used in conjunction with mAbs. DEF201 is an Ad5-IFN-alpha construct. When DEF201 was given to cynomolgus macaques one day after exposure, followed by mAbs at day four, 50% survived. When the DEF 201 and mAbs were given after detection of viremia at day 3, survival was 75% in cynomolgus macaques and 100% in rhesus macaques. These animals survived despite high levels of viremia (10^5^ TCID50 per mL) and developed robust immune responses [34]. Humanized antibodies made in plants or Chinese hamster ovarian cells were also effective. The Public Health Agency of Canada is working with MappBio to optimize mAb combinations. Many new mAb are being evaluated. In response to a question about whether humoral or cell-mediated immunity was more important in animals that survived re-challenge, Dr. Kobinger responded that survival seemed to be correlated with antibody response. 

Lijun Rong (University of Illinois-Chicago) discussed his group’s small molecule inhibitors of viral entry. They have used a high-throughput compound screen to identify small molecules that are active against numerous viruses. The cell-based assay uses pseudotyped defective HIV-1 with the viral glycoprotein of interest [35]. Broad-spectrum inhibitors may be identified by performing high‑throughput screening using glycoproteins of three viruses (MARV, influenza A virus, and Lassa virus) in parallel. They are seeking molecules that block MARV and EBOV. Approximately 14,000 compounds have been screened, and lead compounds with IC_50_ in the range of 1.3 µM have been identified. Some of these have been tested on pseudotyped virus and infectious virus. They also generated initial mouse efficacy data. Structure activity relationships are being evaluated. 

John M. Dye (DoD-USAMRIID) discussed mammalian and plant-derived antibody-based therapies against SUDV. His team has developed 15 mAbs that target GP_1,2_ of SUDV, but do not neutralize EBOV. One antibody, 16F6, gave about 80% inhibition at sub-nanomolar concentration in a plaque-reduction neutralization test. There are currently no adequate mouse models for studying SUDV infections. The wild-type SUDV does not produce mortality or observable morbidity in mice. Attempts to develop a mouse-adapted SUDV strain were not successful. As an alternative to test mAbs, they are using interferon α-β receptor knockout mice which show 30%–60% mortality and significant (25%) weight loss. The 16F6 mAb given at a dose of 100 μg one day post-infection increased survival to 90% and reduced weight loss. In contrast, mice receiving an EBOV-specific mAb experienced no reductions of weight loss or mortality compared to the PBS control group. Dr. Dye’s team plans to produce mAbs in tobacco or NSO cells (a myeloma cell line developed for producing mAbs) and compare them *in vitro* and *in vivo*. Plant-derived 16F6 compares favorably to hybridoma-derived mAb in ELISA and neutralization assays. Small molecule screening and development of human mAbs are also being pursued. In response to questions, Dr. Dye replied that all of his mAbs are available for use in virus identification and quantitation. He indicated that the mice that survive SUDV exposure are immune to re-infection. He has not yet studied 16F6 mAb in other animal species. 

### 3.7. Human Disease Experiences; William Dowling, Chair

This session focused on human disease experiences with the goal of presenting information that might be informative for developers of vaccines and therapeutics.

Bill Dowling gave an update about the FANG Human Sub-group’s activities. This is a new Sub‑group which has not met frequently. It is a relatively small group with 14 people, including scientists and clinicians. Their goal is to generate a comprehensive assessment of filovirus disease in humans to determine the degree of parity with animal models and to identify potential markers that may correlate with disease, both in humans and animal models. The Sub-group will collaborate closely with the Animal Models Sub-group. As a first step the group is conducting a comprehensive literature review. Future discussion topics include disease time course, symptoms and signs and effectiveness of different means of supportive care. They also want to clarify the differences between disease caused by MARV *vs.* EBOV, and disease caused by different ebolaviruses.

Jens H. Kuhn (NIH/NIAID) gave a presentation entitled “Filovirus disease outbreaks in numbers—real and perceived threat differences?” His presentation highlighted the difficulties of obtaining accurate values for the numbers of cases, number of fatalities, and thus case-fatality rates for various disease outbreaks caused by filoviruses. Several interconnected issues contribute to the problem, including which “cases” to count, which is dependent on what defines a ‘case’ and who defines them. Case definitions may be based on clinical signs, patient symptoms, detection of virus, detection of antibodies, recollections of clinicians, or combinations of these and other information. The definition chosen may greatly change the number of reported cases, and this will change the denominator for case-fatality rates, even if the number of fatalities is clear. What determines the basis of attributing death to filovirus infection must also be considered. The number of cases changes as an outbreak proceeds, growing and then leveling off, or even decreasing as data become more definitive. Even after an outbreak is over, the reported number of cases may differ depending on the source of information. During earlier outbreaks, less rigorous criteria may have been used than those used today, which makes it difficult to compare current and historical data. When outbreaks are large, relatively good case number estimates are possible, but for small outbreaks, a variation of a few cases may change conclusions substantially. Comparisons between outbreaks caused by different filoviruses are difficult when the underlying data are uncertain. Dr. Kuhn questioned if our belief about EBOV being more lethal than SUDV is based on reliable data, and whether the number of reported cases in an outbreak can be used to draw conclusions about the virulence of a particular filovirus. Investigators too often focus on defining the properties of a particular filovirus to draw conclusions about its virulence and endemicity and fail to document the many host factors that contribute to case outcomes in outbreaks, such as immune status, presence of co-infections, and host genetics. Cultural factors among afflicted populations may influence how people respond to illness during an outbreak and further confound the quality of the outbreak investigation. Further, many clinical and epidemiological data sets are never published. Consequently, valuable information that may be used to better define case numbers and possibly support conclusions may be inaccessible to the wider community. Improved sharing of information, particularly of unpublished data, would clearly help, but is only one facet of a complicated activity.

Pierre Rollin (CDC) presented “Filovirus outbreaks: field challenges” to highlight the realities of field studies in remote, underdeveloped areas of central Africa [36,37]. Clinicians, researchers, and charitable organizations respond to outbreaks to mitigate public health emergencies, but also to identify outbreak sources, limit dissemination, respond to public concerns, and conduct scientific studies. There is usually a complex group of participants at an outbreak, not all with the same goals; this may include the Ministry of Health of the affected country, WHO, CDC, Médecins Sans Frontières, Health Canada, and others. The politics of outbreak management come into the picture. Logistics are very complex, requiring transportation to bring people, materials, security, personal protective equipment (PPE), electricity, and water, among others. Isolation of patients is desirable to stop transmission, but is complicated by many issues. First is the need for trained people who understand infection control. For example, PPE reduces the ability to care for patients. Field clinical laboratories do not have comprehensive biosafety or technical capabilities; electricity, refrigeration, and essential equipment are not a given. Basic tests such as PCR, ELISA, antigen detection and antibody detection may not always be possible. Data collection in the field is a major challenge; clinical charts may not exist, and records may be burned for infection control. Cultural concerns may preclude collecting samples or hamper efforts such as performing autopsies. Transmission is generally by direct person-to-person contact, particularly among family members and health-care workers. Outbreak tracking is possible but often difficult; numbers change and it takes time and effort to “clean up” data. Challenges include refinement of case definitions and database management. Index cases are often not identified until secondary transmission has occurred. Investigations to identify reservoirs have led to a correlation between human cases of Marburg hemorrhagic fever and exposures to infected bats in a mine in Uganda, but the reservoir has not been definitively determined. Health education in filovirus endemic regions is a challenge. It is difficult to encourage people to access medical care if treatments are not available. Point-of-care diagnostics and improved medical staffing would enhance the utility of potential MCMs. However, there would be major difficulties conducting clinical trials of MCM candidates. For all these reasons, completion of optimal field studies and collection of reliable data is difficult at best. In response to questions, Dr. Rollin said the frequency of outbreaks may be increasing, although this may be due in part to improved recognition of suspect disease and enhanced surveillance in more populated areas. It is possible that small outbreaks in remote areas are not being identified.

### 3.8. Panel Discussion about the Filovirus MCM Pipeline; Ed Nuzum, (NIH/NIAID/DMID), Chair

The final session was a panel discussion with speakers from academia, industry and government who have experience in the areas of therapeutics and vaccines; some have developed filoviruses MCM candidates and some have not. The panelists were asked to comment on: lessons learned; gaps, challenges and bottlenecks; and their perspectives on how filovirus MCM development could be facilitated.

Jean Patterson is director of the BSL-4 laboratory at Texas Biomedical Research Institute. She focused on the gaps that have been identified and are being addressed by the FANG. These include standardized virus assays, well-characterized challenge materials, and consistent animal models. There has been much progress on these issues. Sequencing and known particle-to-pfu ratios are important characteristics of challenge materials, as is passage history. The problem of control animals surviving exposure is probably related to exposure dose. There is still a need for more natural history studies. The lack of well-established euthanasia criteria is another gap, and veterinarians must be involved in formulating standards. However, the community has come a long way, and the use of several different laboratories to validate methods and protocols has been very effective.

Mary Kate Hart has vaccine development experience at USAMRIID and currently at DynPort Vaccine Corp. She indicated that the FANG efforts have been valuable and commented that the biggest gaps for filovirus vaccine development are the identification of correlates of protection and availability of assays. Bridging across animals of different species under the Animal Rule is also a significant challenge. Ideally, correlate assays would be species-neutral to avoid the need to determine how species-specific reagents react relative to each other, which adds complexity and cost to vaccine development. Bridging from animal studies to human trials also raises issues. Animal studies strive for 100% efficacy, but for predicting clinical benefit in humans it may be necessary to change vaccine doses to induce immune responses in the animals that reflect those observed in the clinic. The initiation of clinical trials as soon as possible may enhance the bridging of animal and human studies, and will provide opportunities to obtain early clinical data and samples for correlate assay development. Manufacturing is also a substantial issue, and it is desirable to involve contract manufacturing organizations (CMOs) early, and to communicate the desired final scale of manufacturing as soon as possible. Reducing requirements late in development may negate the results of substantial efforts and funding that were unnecessarily expended, while increased demand may require scale-up in manufacturing with identification of scale-dependent parameters. CMOs need incentives if the post-licensure manufacturing requirements are small. 

Annie Frimm is head of regulatory affairs at SIGA Technologies. She discussed lessons learned during development of their therapeutic tecovirimat which was developed for variola virus and other orthopoxviruses. Usually, animals of multiple species must be studied to gain regulatory approval. The challenge for SIGA was that humans are the only natural hosts of variola virus. They were allowed to use monkeypox and rabbitpox viruses in cognate animal models to generate essential efficacy data. This FDA decision was based on expert consultations and an FDA smallpox advisory committee. Since there are significant logistical challenges to conducting GCP studies of monkeypox in humans in Africa, animal studies are considered the better approach. Extensive and open communication with FDA was the key, and this takes time. To develop products under the Animal Rule, natural history data using modern techniques is important and will support the identification of a therapeutic trigger. Pivotal animal studies should be GLP and as close to GCP as possible, as these studies will be reviewed by both animal and clinical reviewers at FDA. Basing the dose for humans on animal data may lead to high conservative dose estimates.

Thomas Ksiazek is a veterinarian and virologist from UTMB with filovirus experience at overseas Navy research labs, USAMRIID, and CDC. Animal models are critical to the development of MCMs. However, they may pose challenges to regulatory approval. Recent progress on filovirus countermeasures in NHP models is encouraging. Dr. Ksiazek commented that during his time at USAMRIID, there were no countermeasures capable of protecting NHPs from filovirus disease and death. Standardization of filovirus exposure material is also important as differences between exposure viruses are beginning to be apparent. Transmission of these viruses is manageable and the risk is not as great as some think; infection control is the key. The outbreaks themselves are often the result of poor or absent infection control practices in African medical facilities. Using only laboratory-confirmed human cases is important to for understanding the natural history of diseases caused by filoviruses. The logistical challenges of working on sporadic outbreaks in remote locations make field studies difficult. In response to a question about what the use for filovirus vaccines would be, he commented that immunization efforts in animals might be worth pursuing as a means of preventing human disease, but that education about the potential sources of infection and infection control may be more effective.

David Aglow is a contractor for BARDA with over 30 years experience in industry. He currently works on CMC issues. BARDA is generally concerned with Phase 1 and later stages of product development. He shared lessons learned that may help people benefit from others’ difficulties. Process development and scale up are frequently unsuccessful. CMOs often change which may cause technology transfer issues that waste time and money. The choice of CMO is important; the best partner should either be able to complete the project, or be good at transitioning the effort to another organization. It is important to know the consumer for a product since that will impact scale issues and acceptable cost. Timelines need to be realistic; 10 years from Phase 1 to licensure is not unreasonable. Some products may go into the stockpile under an emergency use authorization (EUA) scenario before licensure. But an EUA is not a cure-all; because almost all the same data are needed for final regulatory approval, it may lengthen the time line. GLP and GMP are important for doing things right, not just for satisfying the FDA. Good data matter and it can save time and ultimately money. Studies should be driven to get a product licensed; don’t do costly unnecessary studies.

Ed Nuzum made summary comments about the workshop and emphasized several points. The need for high quality studies in BSL-4 containment laboratories to support development of countermeasures and obtain regulatory approval is a huge task facing product developers. Though FDA cannot require GLP for these studies, GLP-quality work is necessary to generate reproducible and consistent data that engender high levels of confidence. Any product in the pre-clinical stages of development must be supported by data resulting from quality studies. Development of quality models, assays and systems must start early in the development process because so much time is needed to attain them. The time and costs associated with high quality standards may well be recouped in the long run. Regarding FANG activities, he pointed out that the purpose of the Human Sub-group is to provide human disease information which can guide animal model development and animal studies; correlates of protection are an important issue for the Animal Model Sub-group to address in the near future; and though much progress has been made with regard to exposure material, the potential impact of passage number, particle count, *etc.* on virulence is still an evolving story. Electronic data capture and submission to FDA is another topic for future consideration. The partnership between MappBio and Defyrus, two potential competitors, is very impressive. While product development efforts will eventually be successful, there also needs to be a focus on plans for what to do if a filovirus release or laboratory accident were to occur now. For example, efforts to plan for use of mAbs in such scenarios are underway. It was also noted that there are many early products in the pipeline and that we need to identify development gaps and priorities so that we can focus on getting products into clinical testing faster. He commented that we should proceed aggressively and focus on the data needed for IND submissions. Clinical safety and human response data are critically important for product evaluation. Product developers should be pragmatic and move expeditiously towards clinical trials as rapidly as possible. 

### 3.9. Poster Session

A poster session with presentations about product concepts, most in earlier stages of development, was held throughout the workshop, and generated significant interest. The following are the presenters and titles:

#1 Michelle Meyer (University of Texas Medical Branch). Aerosol Vaccination against Ebola Virus
#2 Ronald N. Harty (University of Pennsylvania). Development of Host-Oriented Therapeutics Targeting Hemorrhagic Syndrome Virus Budding
#3 Matthias Schnell (Jefferson Medical College). Antibody Quality and Protection from Lethal Ebola Virus Challenge in Nonhuman Primates Immunized with Rabies Virus Based Bivalent Vaccine
#4 Judith White (University of Virginia). Multiple FDA-Approved Compounds Block Filovirus Infection through an NPC1-Dependent Pathway
#5 Louis Altamura (DoD/USAMRIID). Codon-Optimized Filovirus DNA Vaccines Delivered by Intramuscular Electroporation Protect Cynomolgus Macaques from Lethal Ebola and Marburg Virus Challenges
#6 Pete Halfmann (University of Wisconsin-Madison). Use of a Biologically Contained Ebola Virus as a Platform for Antiviral Discovery and Vaccine Development
#7 Charles D. Murin (Scripps Research Institute). Mapping Antibody Epitopes by Single Particle Electron Microscopy
#8 Ariane Volkmann (Bavarian Nordic GmbH). The Effect of Immune Stimulating Molecules on the Efficacy of Protection Afforded by MVA Based Marburg Vaccines
#9 Julia Biggins (DoD/USAMRIID). Protection of Guinea Pigs from *Zaire ebolavirus* (ZEBOV) Challenge after Vaccination with a SAM^®^ Vaccine Expressing the ZEBOV Envelope Glycoprotein (GP)
#10 Kelly Warfield (Integrated Biotherapeutics, Inc.). Development of a Novel Rationally-Designed Pan-Filovirus Subunit Vaccine
#11 Gerardo Kaplan (FDA/CBER). Development of a Candidate Filovirus Vaccine Based on Trimeric Glycoprotein Fused to Fc of Human IgG1
#12 Sean Amberg (SIGA Technologies). Screening and Characterization of Candidate Filovirus Antivirals
#13 Andrew Flyak (Vanderbilt University). Isolation of Naturally-Occurring Human Neutralizing Monoclonal Antibodies Against Marburg Virus
#14 James Cunningham (Harvard Medical School). Small molecule inhibitors of Ebola virus and Lassa fever virus infection

## Supplementary Files

Supplementary File 1 Supplementary (ZIP, 669 KB)
