# Supplementary material for: Challenges, Progress, and Opportunities: Proceedings of the Filovirus Medical Countermeasures Workshop
Source: Viruses. 2014 Jul 9;6(7):2673–97. doi: 10.3390/v6072673 (PMC4113787; doi:10.3390/v6072673)
Supplement: Supplementary File 1 — Supplementary (ZIP, 669 KB) [file viruses-06-02673-s001.zip › viruses-55705-supplementary files/Abstract - filovirus MCM workshop 2013 8-9-13 RH.PDF]

Filovirus MCM Workshop Poster Presentation Abstracts

Codon-optimized filovirus DNA vaccines delivered by intramuscular electroporation protect cynomolgus macaques from lethal Ebola and Marburg virus challenges

Louis Altamura1*, Rebecca Grant-Klein1, Catherine Badger1, Callie Bounds1, Nicole Van Deusen1, Drew Hannaman2, Lesley Dupuy1, and Connie Schmaljohn1
1 United States Army Medical Research Institute of Infectious Diseases, Frederick, MD
2 Ichor Medical Systems, San Diego, CA

* presenting author, email: Louis.Altamura@us.army.mil

We evaluated the immunogenicity and protective efficacy of DNA vaccines expressing the codon-optimized glycoprotein (GP) genes of filoviruses when delivered by intramuscular electroporation using the TriGrid™ delivery system to cynomolgus macaques. Non-human primates (NHPs) were vaccinated with DNA plasmids expressing genes of Ebola virus (EBOV), Marburg virus (MARV), or a mixture of the EBOV, MARV, Sudan virus and Ravn virus GP DNA vaccines. Control NHPs were vaccinated with the plasmid with no insert. All NHPs receiving the filovirus DNA vaccines developed glycoprotein-specific IgG antibodies, with no significant differences between single and multiagent groups. Most NHPs that received the EBOV or multiagent vaccines developed neutralizing antibodies to EBOV (5/6 vs 6/6). Those NHPs that received the MARV vaccine all developed neutralizing antibodies (6/6), but most animals that received the multiagent vaccine failed to do so (1/6). Studies are in progress to assess the pre-challenge CD4(+) and CD8(+) T-cell memory responses to each virus. A greater proportion of NHPs vaccinated with the EBOV vaccine survived lethal EBOV challenge in comparison to NHPs that received the multiagent vaccine (5/6 vs 1/6), suggesting immunological interference. In contrast, both the MARV and multiagent vaccines were able to protect NHPs from lethal MARV challenge (5/6 vs 6/6). These data provide evidence that codon-optimized DNA vaccines against filoviruses can elicit protective immune responses in NHPs and that total IgG and neutralizing antibody responses are poor indicators of protection. Furthermore, additional studies will be required to identify and overcome potential interference issues with filovirus multiagent vaccines.

Protection of guinea pigs from Zaire ebolavirus (ZEBOV) challenge after vaccination with a SAM® vaccine expressing the ZEBOV envelope glycoprotein (GP)

Julia Biggins1*, Armin Hekele2, Luis Brito2, Peter Mason2, Clayton Beard2, Jeff Ulmer2, Andrew Geall2, and Gene Olinger1
Affiliation:

1Divison of Virology, USAMRIID, Frederick, MA 21702, USA.
2Novartis Vaccines and Diagnostics, Inc. (NV&D), Cambridge, MA 02139, USA.
*Poster presenter, Julia Biggins: julia.e.biggins.ctr@mail.mil

Ebola viruses are enveloped, negative single-stranded RNA viruses with a genome of 19 kb, which belong to the filoviridae family. It is one of the most deadly human pathogens, causing up to 90% mortality among infected individuals. There are no licensed vaccines or treatments available, but several promising viral vector-based vaccine candidates are under development, including an alphavirus-

based viral replicon particle (VRP) system. Most of these vaccines have expressed the ebola virus glycoprotein (GP) because it can induce both antigen-specific cellular and humoral immune responses. The SAM vaccine platform technology offers a unique cell-free production capability, avoiding the considerable safety and scale-up issues faced by viral vector production in cell culture. The use of a non-viral delivery system also avoids potential issues of anti-vector immunity faced by viral vectors and allows a relatively low dose of RNA to be used when compared to unformulated RNA. As part of a research collaboration between USAMRIID and NV&D, an Ebola SAM vaccine was produced. The Zaire Ebola-GP sequence was cloned into the SAM vector, and was formulated with a cationic nanoemulsion (CNE) or lipid nanoparticle (LNP) delivery system and tested in a guinea pig challenge model. The Ebola SAM (GP/CNE/37.5 µg) and Ebola SAM (GP/LNP/15 µg) vaccines elicited GP-specific antibodies that were protective (100%) relative to the PBS negative control (0%) and were equivalent to the VRP positive control. Future studies will focus on evaluation of the vaccines in a non-human primate challenge model.

Aerosol Vaccination against Ebola Virus

M. Meyer1*, T. Garron1, G. Olinger5, C. Mire2, C. Klages4, T. Geisbert2, P. Collins6, A. Bukreyev1,2,3. 1Departments of Pathology and 2Microbiology & Immunology, 3Sealy Center for Vaccine Development, 4Galveston National Laboratory, The University of Texas Medical Branch, Galveston, Texas; 5Viral Pathogenesis and Immunology Branch, Virology Division, United States Army Institute for Infectious Diseases, Frederick, Maryland; 6National Institute of Allergy and Infectious Diseases, Bethesda, NIH, Maryland.

Previously, we demonstrated two doses of a mucosal human parainfluenza virus type 3-vectored vaccine expressing the glycoprotein (GP) of EBOV (HPIV3/EboGP), delivered as a liquid via the combined intranasal/ intratracheal (IN/IT) route, generated a robust neutralizing antibody response and provided 100% protection of Rhesus macaques against death and disease caused by a highly lethal dose of EBOV challenge virus. Here we vaccinated Rhesus macaques with an aerosolized form of the vaccine, which represents an ideal and feasible means of delivery never been tested for hemorrhagic fever viruses. Groups of macaques received the (i) HPIV3/EboGP vaccine, delivered to the respiratory tract as a small particle (2.5–4.0 µM) aerosol using an AeronebLab nebulizer, or (ii) as a liquid, (iii) Venezuelan equine encephalitis virus replicon vaccine expressing EBOV GP, delivered by the intramuscular route, previously shown to protect macaques against EBOV and (iv) the control empty HPIV3 vector via the IN/IT route. The EBOV-specific IgG and IgA and EBOV-neutralizing serum antibody responses to aerosolized HPIV3/EboGP were equal to or exceeded that observed in the group, which received the liquid form. Given robust neutralizing antibody titers were generated after the first aerosol dose, a challenge study in a new cohort of Rhesus macaques was conducted, revealing both single or booster administration of the aerosolized vaccine conferred 100% protection against death and severe disease caused by a highly lethal dose of EBOV.

Development of a candidate Filovirus vaccine based on trimeric glycoprotein fused to Fc of human IgG1

AUTHORS

Gerardo Kaplan*, Siham Nakamura, Bradford Hilson, Jerome Jacques, and Krishnamurthy Konduru CBER – FDA Email: gerado.kaplan@fda.hhs.gov

ABSTRACT
Filovirus vaccines based on the viral glycoprotein (GP) expressed in viral vectors, such as adenovirus or vesicular stomatitis virus, or as virus like particles are highly effective in the Filovirus mouse, guinea pig, and nonhuman primate (NHP) lethal challenge models. However, initial vaccine studies using purified GP expressed in the baculovirus system yielded disappointing results. We have recently shown that the ebolavirus GP fused to the Fc fragment of human IgG1 (EBOVgp-Fc), which resembles the trimeric structure of GP expressed at the virus and cell surface, conferred protection against ebolavirus in the mouse (Konduru et al., 2011) and guinea pig (Konduru et al., manuscript in preparation) challenge models. We hypothesize that EBOVgp-Fc will also elicit cellular, humoral, and neutralizing immune responses in NHPs and confer protection against ebolavirus lethal challenge. To test this hypothesis, we immunized 2 NHPs i.m. with 0.4 mg of EBOVgp-Fc adjuvanted with poly-IC (4 mg) using a primary inoculation followed by 3 boosts. The NHPs developed high titers of total (1:80,000) and neutralizing antibodies (1:800) against GP, and a strong CD8+ T-cell recall response against GP. The results of an ongoing dose-response immunogenicity study and a small challenge study in NHPs using 0.05 mg of EBOVgp-Fc will be discussed at the Workshop. Our studies showed that EBOVgp-Fc elicited cellular, humoral, and neutralizing responses in NHPs commensurate with protection against Filovirus lethal challenge. Further studies in NHPs will be required to determine whether the EBOVgp-Fc vaccine could be further developed as a candidate Filovirus vaccine.

The Effect of Immune Stimulating Molecules on the Efficacy of Protection Afforded by MVA Based Marburg Vaccines

Ariane Volkmann*, Cédric Cheminay, Yvonne Wollmann, Paul Chaplin
Bavarian Nordic GmbH, Martinsried, Germany
*ariane.volkmann@bavarian-nordic.com

Bavarian Nordic has generated several vaccine candidates for the protection against Marburg virus (MARV) induced hemorrhagic fever disease. The recombinant attenuated live virus vaccine candidates are based on Modified Vaccinia Ankara (MVA-BN®), a third generation smallpox vaccine that has been shown to be safe also in immunocompromised people and the elderly and that is currently being stored in the U.S. Strategic National Stockpile.

Two of the vaccine candidates, MVA-BN® expressing either the glycoprotein (GP) from MARV (MVA-BN® MARV) alone or also expressing GP from Ebola Zaire and Sudan (MVA-BN® Multifilo), have been GMP manufactured and demonstrated to be well tolerated in a repeated dose toxicity and local tolerance study in rabbits. In NHP, MVA-BN® Multifilo provided complete protection in two out of three MARV challenge studies. It was also 100% protective against Ebola Zaire challenge in mice, and partially protective in the NHP model.

In an effort to enhance the immunogenicity of MARV GP, the immune stimulatory molecules CD40L, IL-15, TRICOM or TTC as fusion protein have been cloned into MVA-BN® MARV. Preliminary results in NHP demonstrate improved protection against MARV challenge afforded by co-expression of CD40L. Furthermore, TRICOM enhanced MVA backbone induced protection against monkeypox virus challenge.

Improving the onset and overall immunogenicity of the recombinant MARV GP by co-expression with immune stimulating molecules increases the potential utility of a Marburg vaccine in a post-event setting, while potentially at the same time improving the on-set of protection against smallpox.

The work was supported by NIAID.

Screening and Characterization of Candidate Filovirus Antivirals

Cinthia Costas Jones,[1] Amy Moore,[1] Dima Gharaibeh,[1] Natasha Cerutti,[1] Dennis Hruby,[1] Peter Halfmann,[2] Robert Allen,[1] Sean Amberg[1]*

Utilizing a Zaire ebolavirus minigenome reporter assay (Jasenosky et al, 2010), as well as pseudotyped virus assays employing the Zaire ebolavirus or Marburg marburgvirus envelope proteins, SIGA completed primary screening of our 250K small molecule compound library in an effort to identify inhibitors or filovirus replication. Identified hit compounds were further tested across a range of concentrations for in vitro efficacy in pseudotyped virus or minigenome assays as well as for cytotoxicity, and EC50 and CC50 values for each candidate were calculated. Compounds with a selective index (SI, CC50/EC50) > 10 were profiled for spectrum of antiviral activity and in vitro ADME properties. Candidate compounds are being evaluated for antiviral efficacy using the [illegible]VP30 Zaire ebolavirus system in the Kawaoka Lab at the University of Wisconsin. Candidate compound tolerability and pharmacokinetics in mice is currently being determined prior to evaluation of compound efficacy in a lethal murine model of Zaire ebolavirus virus infection.

1SIGA Technologies, Corvallis, OR,
2Influenza Research Institute, Department of Pathological Sciences, School of Veterinary Medicine, University of Wisconsin-Madison, Madison, WI,
*email: samberg@siga.com

Multiple FDA-Approved Compounds Block Filovirus Infection Through an NPC1-Dependent Pathway.

J.M. White*[1,2], C.J. Shoemaker[1], K.L. Schornberg[1], S.E. Delos[1], C. Scully[3], J.M. Brannan[3], A. Stossel[3], C. Lear[3], L.E. DeWald[3], L.E. Hensley[3], G.G. Olinger[3], B.G. Hoffstrom[4], H. Pajouhesh[4], J. Lehár[4] and L.M. Johansen[4]. Depts. of Cell Biology[1] and Microbiology[2], University of Virginia, Charlottesville, VA, USA; U.S. Army Medical Research Institute of Infectious Diseases (USAMRIID)[3], Fort Detrick, MD, USA; Zalicus Inc.[4], Cambridge, MA, USA. *jw7g@virginia.edu

Filoviruses cause lethal hemorrhagic fevers in humans and nonhuman primates. Moreover, due to a lack of approved therapeutics they remain serious health and biodefense concerns. From a library containing FDA-approved drugs, we identified ~130 compounds that inhibit replication of Zaire ebolavirus (EBOV). Two of the most active FDA-approved compounds, clomiphene and toremifene, were also shown to inhibit replication of Sudan ebolavirus, Marburgvirus, and Ravn virus (in tissue culture). Importantly, clomiphene and toremifene (given 1 hr post infection) significantly enhanced survival of mice challenged with mouse adapted EBOV. Studies with viral-like particles (VLPs) indicated that clomiphene and toremifene block EBOV glycoprotein (GP)-mediated fusion with late endosomes (LE).
Clomiphene and toremifene are cationic amphiphillic drugs (CADs), a class of small molecules that also includes U18666A. U18666A is known to induce dysfunctions associated with loss of Niemann-Pick C1

(NPC1), a LE membrane protein that is essential for EBOV entry1,2. We found that (like U18666A) clomiphene and toremifene (and five other CADs tested) caused cholesterol accumulation in LE. Moreover, higher concentrations of these CADs were needed to inhibit EBOV GP-mediated infection of cells overexpressing NPC1 vs. parental cells. The CADs did not, however, block binding of EBOV GP to NPC1.

Collectively our findings indicate that clomiphene, toremifene, and other CADs block EBOV entry through an NPC1-dependent pathway. Importantly our work has revealed multiple FDA-approved CADs, notably clomiphene and toremifene, as promising anti-filoviral agents, and has highlighted the general approach of screening FDA-approved compounds for drugs that can be repurposed for use against deadly pathogens.

The work was supported by NIH grant U54 AI57168, The Joint Science & Technology Office for Chemical & Biological Defense project CBCALL12-VAXV2-1-0756, and DOD subcontracts W81XWH-08-0051 and W81XWH-12-P-0007.

References:

1J. E. Carette et al., 2011. Nature 477, 340.
2M. Cote et al., 2011. Nature 477, 344.

Antibody Quality and Protection from Lethal Ebola Virus Challenge in Nonhuman Primates Immunized with Rabies Virus Based Bivalent Vaccine

Joseph E. Blaney1$, Andrea Marzi2$, Mallory Willet3$, Amy B. Papaneri1, Christoph Wirblich3, Friederike Feldmann2, Michael Holbrook4, Peter Jahrling1,4, Heinz Feldmann2, Matthias J. Schnell3,5,*

* Equally contributing
1Emerging Viral Pathogens Section, National Institute of Allergy and Infectious Diseases, National Institutes of Health, Bethesda MD, 20892 USA

2Laboratory of Virology, Division of Intramural Research, National Institute of Allergy and Infectious Diseases, National Institutes of Health, Hamilton MT, 59840 USA

3Department of Microbiology and Immunology, 5Jefferson Vaccine Center, Jefferson Medical College, Thomas Jefferson University, Philadelphia PA, 19107 USA

4Integrated Research Facility, National Institute of Allergy and Infectious Diseases, National Institutes of Health, Fort Detrick MD, 21702 USA

Abstract
We have previously described the generation of a novel Ebola virus (EBOV) vaccine platform based on (a) replication-competent rabies virus (RABV), (b) replication-deficient RABV, or (c) chemically inactivated RABV expressing EBOV glycoprotein (GP). Mouse studies demonstrated safety, immunogenicity, and protective efficacy of these live or inactivated RABV/EBOV vaccines. Here we evaluated these vaccines in nonhuman primates. Our results indicate that all three vaccines do induce potent immune responses against both RABV and EBOV, while the protection of immunized animals against EBOV was largely dependent on the quality of humoral immune response against EBOV GP. We also determined if the induced antibodies against EBOV GP differ in their target, affinity, or the isotype.

Our results show that IgG1-biased humoral responses as well as high levels of GP-specific antibodies were beneficial for the control of EBOV infection after immunization. These results further support the concept that a successful EBOV vaccine needs to induce strong antibodies against EBOV. We also showed that a dual vaccine against RABV and filoviruses is achievable; therefore addressing concerns for the marketability of this urgently needed vaccine.

Development of a novel rationally-designed pan-filovirus subunit vaccine

Kelly L. Warfield*1, Frederick W. Holtsberg 1, Sergey Shulenin1, Sven Enterlein1, Hong Vu1, Thomas Geisbert2, and M. Javad Aman 1

1 Integrated Biotherapeutics, Inc. Gaithersburg, MD, USA; 2 University of Texas Medical Branch, Galveston, TX, USA
* kelly@integratedbiotherapeutics.com

The filoviruses, Ebola (EBOV) and Marburg (MARV), are enveloped, filamentous viruses belonging to the Filoviridae family. Most known human infections with these viruses have been fatal and no vaccines or effective therapies are currently available for use in humans. EBOV and MARV glycoproteins (GP) mediate the viral entry into host cells. There are currently no filovirus vaccines available for human use and a major challenge is the development of a vaccine capable of protecting a wide range of the known and emerging viral species. We hypothesized that designing a vaccine targeting immune responses to the highly conserved receptor-binding region (RBR) of the filovirus GP could generate a simple and efficacious pan-filovirus vaccine. Based on the fact that the RBR is known to be masked by the bulky mucin-like domain, we have generated subunit proteins of GP with the mucin-like domain (MLD) deleted (GPddMuc). Serological ELISAs using the GPddmuc proteins have shown that immune responses to these proteins can correlate with protection of nonhuman primates against lethal filovirus infections. The GPddmuc proteins, used as vaccines, induced strong antibody responses in mice, guinea pigs and nonhuman primates. Proof-of-concept protection studies in mice have been completed and studies in guinea pigs are currently under way to compare protection induced by the GPddmuc proteins with full-length soluble GP proteins produced using a baculovirus-insect cell system.

Development of Host-Oriented Therapeutics Targeting Hemorrhagic Syndrome Virus Budding

Jianhong Lu1, Ziying Han1, Yuliang Liu1, Gordon Ruthel1, Bruce D. Freedman1, Michael S. Lee2,3, Mark A. Olson2, and Ronald N. Harty1*

1Department of Pathobiology, School of Veterinary Medicine, University of Pennsylvania, 3800 Spruce St., Philadelphia, PA 19104; 2Integrated Toxicology Division and 3Computational Sciences Division, U.S. Army Medical Research Institute of Infectious Diseases (USAMRIID), 1425 Porter St., Fort Detrick, MD 21702.

There is an urgent need for the development of safe and effective therapeutics against biodefense and NIAID Category A pathogens, including filoviruses (Ebola and Marburg) and arenaviruses (e.g. Lassa and Junín) which cause severe hemorrhagic fever syndromes with high mortality rates. We and others have established that efficient budding of filoviruses and arenaviruses is critically dependent on the subversion of host proteins such as Tsg101, and that PTAP late (L) budding domains expressed by these

viruses are critical for mediating such interactions. As disruption of budding would prevent virus dissemination, identification and optimization of small molecule inhibitors that block these critical viral-host interactions should also effectively block disease progression and transmission. We have used the NMR structure of the Tsg101-PTAP interaction site to guide the in silico selection and design of competitive protein interaction blockers. Promising preliminary results for select lead compounds have been obtained using functional VLP budding assays, live virus infection of cells in culture, high-resolution imaging, and bimolecular complementation assays of virus-host interactions in live mammalian cells. Together, these data demonstrate that inhibitors we have identified are specific and that this approach is feasible. Indeed, nanomolar concentrations of our lead candidate: i) inhibited Ebola and Junín VLP egress, ii) inhibited egress of the live Candid-1 vaccine strain of Junín virus in cell culture, and iii) specifically blocked the PTAP-Tsg101 virus-host interaction in live mammalian cells. We plan to move our lead candidate broad-spectrum therapeutic into proof-of-concept efficacy studies in appropriate animal models and complete preclinical IND-enabling virology studies.

* rharty@vet.upenn.edu

Use of a Biologically Contained Ebola Virus as a Platform for Antiviral Discovery and Vaccine Development

Peter Halfmann1*, Andrea Marzi2, Hideki Ebihara2, Hideyuki Saya3, Heinz Feldmann2 and Yoshihiro Kawaoka1,4,5

1Influenza Research Institute, Department of Pathobiological Sciences, School of Veterinary Medicine, University of Wisconsin-Madison, 575 Science Drive, Madison, WI 53711, USA.

2DIR, NIAID, NIH, Rocky Mountain Laboratories, 903 South 4th Street, Hamilton, MT 59840 USA.

3Division of Gene Regulation, Institute for Advanced Medical Research, School of Medicine, Keio University, 35 Shinano-machi, Shinjuku-ku, Tokyo 160-8582, Japan.

4Division of Virology, Department of Microbiology and Immunology, Institute of Medical Science, University of Tokyo, Tokyo 108-8639, Japan.

5Department of Special Pathogens, International Research Center for Infectious Diseases, Institute of Medical Science, University of Tokyo, Minato-ku, Tokyo 108-22 8639, Japan.
* halfmanp@svm.vetmed.wisc.edu

Previously, we established a biologically contained Ebola virus (EbolaΔVP30 virus) that lacks the essential viral VP30 gene and can be maintained only in cells that stably express this gene. EbolaΔVP30 virus is a BSL-3 agent that grows with similar kinetics and titers to those of authentic Ebola virus. Therefore, EbolaΔVP30 virus is an ideal surrogate to study authentic Ebola virus.
A variant of EbolaΔVP30 virus contains the reporter gene Renilla luciferase (ren-luc), making it a useful and efficient tool for high-throughput screens. Suppression of ren-luc expression in the presence of inhibitor compounds is a reliable and robust read-out for high-throughput screens of small molecule compound libraries. This system can thus be used to efficiently identify inhibitors of any step in the virus life cycle, a clear advantage over single-step assays.
As a whole virus vaccine candidate, EbolaΔVP30 virus confers protection in immunized animals against a lethal challenge of Zaire Ebola virus in experimental models such as mice, guinea pigs, and nonhuman

primates. Protection of animals immunized with EbolaΔVP30 virus was associated with a B-cell and T-cell immune response to the vaccine virus.
Here, we describe the value of this biologically contained Ebola virus for the discovery and development of therapeutics to combat filovirus infections.

Isolation of naturally-occurring human neutralizing monoclonal antibodies against Marburg virus

Andrew Flyak1*, Philipp Ilinykh3,4, Andrew Ward5, C. Daniel Murin5, Takao Hashiguchi6, Marnie Fusco6, Erica Ollmann Saphire6,7, Tania Garron3,4, Thomas G. Ksiazek3,4, Alexander Bukreyev3,4, and James E. Crowe, Jr.1,2

1Department of Pathology, Microbiology, and Immunology, 2Vanderbilt Vaccine Center, Vanderbilt University, Nashville, TN, 3Department of Pathology, University of Texas Medical Branch, Galveston, TX, 4Galveston National Laboratory, Galveston, TX, 5Department of Integrative Structural and Computational Biology, 6Dept. of Immunology and Microbial Science and 7The Skaggs Institute for Chemical Biology of The Scripps Research Institute, La Jolla, CA

*presenting (andrew.i.flyak@vanderbilt.edu)

Marburg (MARV) virus infects humans and nonhuman primates causing hemorrhagic fever with mortality rates up to 90%. MARV infections cause local outbreaks in Sub-Saharan Africa, and are classified as category A select agents. Currently no approved treatments against filoviruses are available. Recent studies have suggested that antibody-mediated protection against filoviruses is achievable.

We isolated over 50 human antibodies against MARV proteins using peripheral blood B cells from a single U.S donor who survived MARV infection several years earlier. We used recombinant MARV proteins as well as irradiated MARV virions to screen supernatants of transformed B cells and made human hybridomas from cell lines that secreted MARV-specific antibodies. We screened antibodies in a neutralization assay and identified several antibodies that showed potent neutralization activity. In order to define the basis of neutralization we have obtained a low resolution EM structure of one neutralizing antibody (MR78) bound to GP and determined that MR78 binds to MARV GP near the receptor binding site. We have generated a MARV glycoprotein-pseudotyped vesicular stomatitis virus (VSV-GP) escape mutant for the antibody MR78, which is resistant to neutralization by the antibody. The mutant has a single substitution in GP, indicating the location of the epitope: Ala214Val. Collectively, these data demonstrate the feasibility of isolation of neutralizing human monoclonal antibodies against MARV using B cells obtained from individuals who survived MARV infection. The information obtained in our study can be used to develop new therapeutics and structure-based vaccine design against filoviruses.

Mapping Antibody Epitopes by Single Particle Electron Microscopy

Charles D. Murin1,2, Marnie Fusco1, Takao Hashiguchi1, Andrew Flyak3, James Crowe3, Alex Bukreyev4, Gene Olinger5, Larry Zeitlin6, John M. Dye5, Erica Ollmann Saphire1,7, Andrew Ward2

1Department of Immunology and Microbial Science, The Scripps Research Institute, La Jolla, CA
2Department of Integrative and Structural Biology, The Scripps Research Institute, La Jolla, CA

3Department of Pathology, Microbiology and Immunology, Vanderbilt Medical Center, Nashville, TN
4University of Texas Medical Branch, Galveston, TX
5 Division of Virology, United States Army Medical Research Institute for Infectious Disease
6Mapp Bio, Inc., San Diego, CA
7The Skaggs Institute for Chemical Biology, The Scripps Research Institute, La Jolla, CA

Antibody cocktails have recently proven to be efficacious in post-exposure treatment of filovirus infection in both rodents and nonhuman primates. The current goal is to identify and optimize the most effective antibody cocktail(s). However, it is imperative that we understand how and where each antibody binds, so that a cocktail of greatest synergy and ideally, cross-reactivity, can be developed. In pursuit of this goal, we are first mapping antibody epitopes on the surface of Ebola virus GP trimers using single particle electron microscopy, in parallel with X-ray crystallography. We have begun mapping the epitopes of three antibodies which have already proven effective in protecting rhesus macaques from infection taken pre- or post-exposure, namely two mAbs developed at USAMRIID against the mucin-like domain of Ebola virus GP (6D8 and 13F6) and one mAb that recognizes an epitope shared between GP and sGP (13C6). We have also begun mapping epitopes for mAbs against Marburg virus GP, which promise to aid in crystallization of the as of yet un-resolved structure. By mapping a large number of protective/neutralizing antibody epitopes by negative stain reconstructions, we aim to quickly categorize the repertoire of existing antibodies. This work, plus further, detailed characterization by cryo-EM and X-ray crystallography will allow correlation of recognized epitope with functional efficacy in order to identify the most effective antibody cocktail.

S **Small molecule inhibitors of Ebola virus and Lassa fever virus infection.**

James M. Cunningham MD, Departments of Medicine/Microbiology, Brigham and Women’s Hospital and Harvard Medical School, Boston MA.

A major focus of research in my laboratory is to identify small molecule inhibitors of viral pathogens and to determine their mechanism of action and their potential for development into anti-viral drugs. To this end, we have developed cell-based assays to screen small molecule libraries for inhibitors of infection and new methods to identify the targets of these inhibitors in cells. In recently published studies, we reported the identification of an adamantyl benzyl piperazine compound that is a potent inhibitor of Ebola virus (EboV) infection (IC_{50}<20 nM). We found that the target of this inhibitor is the lysosome membrane protein Niemann-Pick C1 (NPC1) and showed that the inhibitor interferes with the essential function of NPC1 as the EboV glycoprotein receptor. The molecular basis for the anti-EboV activity of this compound is analogous to that of the FDA approved HIV drug maraviroc. Importantly, we observe that NPC1 is essential for infection by virus isolates from all species of *Filoviridae* including Marburg virus. We have now devised solution-based assays to analyze NPC1 receptor function and are employing these assays to inform structural studies and analysis of inhibitor resistance. An additional major effort is directed at a second compound identified in the screen that is also a nanomolar inhibitor of EboV and also SARS-CoV infection. Our studies indicate the target of this inhibitor is a host protein required for the trafficking of virus particles to lysosomes in cells and we are now intensively studying the role of this protein in infection and in inhibitor action. We have also screened for inhibitors of Lassa

fever virus infection and are using the methods developed to analyze EboV inhibitors to identify the cell target of a promising lead compound obtained by extensive SAR. Our expectation is that the well-characterized inhibitors identified in these studies are strong candidates for further development using medicinal chemistry/*in vivo* testing. We are aided in this work by important collaborations with chemists Kyungae Lee and Nathanael Gray (Harvard), virologists Gene Olinger (USAMRIID) and Christina Spiropoulou (CDC), and also support from NERCE.

Supported to R01s AI067927 and AI089498 to E.O.S.
