# Supplementary material for: Challenges, Progress, and Opportunities: Proceedings of the Filovirus Medical Countermeasures Workshop
Source: Viruses. 2014 Jul 9;6(7):2673–97. doi: 10.3390/v6072673 (PMC4113787; doi:10.3390/v6072673)
Supplement: Supplementary File 1 — Supplementary (ZIP, 669 KB) [file viruses-06-02673-s001.zip › viruses-55705-supplementary files/FV workshop agenda final.pdf]

Agenda

Filovirus Medical Countermeasures (MCM) Workshop

August 22-23, 2013

FISHERS LANE CONFERENCE CENTER
Terrace Level
5635 Fishers Lane
Rockville, MD 20852

https://respond.niaid.nih.gov/conferences/MCMWorkshop/Pages/default.aspx

To view the live webcast go to http://videocast.nih.gov/ and look under Today's Events or search the archives after the workshop is over

Workshop Goals

- Presentations by government agencies of their current MCM requirements
- Presentations by selected companies, investigators and sponsors of their candidate MCMs
- Discussion of recent human disease experiences that may inform MCM development
- FANG (Filovirus Animal Non-Clinical Group) sub-group updates regarding assays, challenge material, and animal models for MCM development
- Presentations on conduct of adequate and well-controlled BSL4 studies during MCM development

Thursday, August 22 Morning Session

8:30 Welcome and workshop overview, Ed Nuzum (NIH/NIAID/DMID) and Nicole Kilgore (DoD/MCS-JVAP)

FANG sub-group updates: accomplishments, current and near term project status, lessons learned

Chair, Rebecca Kurnat (DoD/MCS-JVAP)

8:45 Assays, Rebecca Kurnat (DoD/MCS-JVAP)
9:05 Challenge materials, Lucy Ward (NIH/NIAID/DMID)
9:25 Animal models, William Dowling (NIH/NIAID/DMID)

9:45 Networking break and poster viewing

Conduct of adequate and well-controlled BSL4 studies during MCM development

Chair, Lisa Hensley (NIH/NIAID/IRF)

10:15 Andrea Powell (FDA/CDER/OCTEC)
FDA's Expectations Regarding Data Quality and Integrity for the Animal Rule-Specific Studies

10:35 GLP BSL-4: Panel discussion about lessons learned and experiences
Louise Pitt (DoD/USAMRIID)
Ricardo Carrion (Texas Biomedical Research Institute)
Trevor Brasel (University of Texas Medical Branch)

Government agency requirements for MCM

11:30 Nicole Kilgore (DoD/MCS-JVAP) and George Christopher (DoD/MCS-BDT)

11:45 Christopher van de Wetering (HHS/ASPR/OPP/MCSR)

12:00 Lunch and poster viewing

Thursday, August 22 Afternoon Session

Vaccine MCM: presentations by sponsors of vaccine candidates

Chairs, Lucy Ward (NIH/NIAID/DMID) and Pat Repik (NIH/NIAID/DMID)

1:00 Sina Bavari (DoD/USAMRIID/MCS-JVAP)
Virus-Like Particle vaccine for filoviruses

1:20 Gene Olinger (DoD/USAMRIID/MCS-JVAP)
VEE Replicon Particle Vaccine for Filoviruses

1:40 Benoit Callendret (Crucell Holland BV/Janssen Pharmaceuticals)
Advanced Development of a Multivalent Filovirus Hemorrhagic Fever Vaccines Based on Ad26 and Ad35 Recombinant Adenoviral Vectors

2:00 Alfredo Nicosia (Okarios/GSK)
Novel filovirus vaccines based on clinically validated Chimpanzee Adenovirus and MVA vector technology

2:20 Networking break and poster viewing

2:50 John H. Eldridge (Profectus BioSciences)
rVSV Vectored Vaccine to Protect Against Ebola and Marburg Viruses

3:10 Maria Croyle (University of Texas at Austin)
Evaluation of Protective Immunity after Mucosal Vaccination Against Ebola Virus with an Adenovirus 5-Based Vaccine

3:30 Axel Lehrer (University of Hawaii)
Recombinant Filovirus Antigens are Safe and Potent Immunogens for Inducing Cellular and Humoral Immunity in Rodents and Non-Human Primates and Provide Protection Against Lethal Live Virus Challenge

3:50 Jay Ramsey (NewLink Genetics)
BPSC1001: Post-Exposure and Pre-Exposure Prophylaxis for Ebolavirus using the VSV-ΔG Platform

4:10 Poster viewing and networking

Poster Presenters:

#1 Michelle Meyer (University of Texas Medical Branch)
Aerosol Vaccination against Ebola Virus

#2 Ronald N. Harty (University of Pennsylvania)
Development of Host-Oriented Therapeutics Targeting Hemorrhagic Syndrome Virus Budding

#3 Matthias Schnell (Jefferson Medical College)
Antibody Quality and Protection from Lethal Ebola Virus Challenge in Nonhuman Primates Immunized with Rabies Virus Based Bivalent Vaccine

#4 Judith White (University of Virginia)
Multiple FDA-Approved Compounds Block Filovirus Infection through an NPC1-Dependent Pathway

#5 Louis Altamura (DoD/USAMRIID)
Codon-Optimized Filovirus DNA Vaccines Delivered by Intramuscular Electroporation Protect Cynomolgus Macaques from Lethal Ebola and Marburg Virus Challenges

#6 Pete Halfmann (University of Wisconsin-Madison)
Use of a Biologically Contained Ebola Virus as a Platform for Antiviral Discovery and Vaccine Development

#7 Charles D. Murin (Scripps Research Institute)
Mapping Antibody Epitopes by Single Particle Electron Microscopy

#8 Ariane Volkmann (Bavarian Nordic GmbH)
The Effect of Immune Stimulating Molecules on the Efficacy of Protection Afforded by MVA Based Marburg Vaccines

#9 Julia Biggins (DoD/USAMRIID)
Protection of Guinea Pigs from *Zaire ebolavirus* (ZEBOV) Challenge after Vaccination with a SAM® Vaccine Expressing the ZEBOV Envelope Glycoprotein (GP)

#10 Kelly Warfield (Integrated Biotherapeutics, Inc.)

Development of a Novel Rationally-Designed Pan-Filovirus Subunit Vaccine

#11 Gerardo Kaplan (FDA/CBER)
Development of a Candidate Filovirus Vaccine Based on Trimeric Glycoprotein Fused to Fc of Human IgG1

#12 Sean Amberg (SIGA Technologies)
Screening and Characterization of Candidate Filovirus Antivirals

#13 Andrew Flyak (Vanderbilt University)
Isolation of Naturally-Occurring Human Neutralizing Monoclonal Antibodies Against Marburg Virus

#14 James Cunningham (Harvard Medical School)
Small molecule inhibitors of Ebola virus and Lassa fever virus infection

Friday, August 23 Morning Session

<u>Therapeutic MCM: presentations by sponsors of therapeutic candidates</u>

Chair, Helen Schiltz (NIH/NIAID/DMID)

8:30 Ian MacLachlan (Tekmira Pharmaceuticals Corp.)
Development of Lipid Nanoparticle siRNA-based Therapeutics for Hemorrhagic Fever Virus Infection

8:55 Jay Charleston (Sarepta Therapeutics)
AVI-7288: A Versatile Antiviral for Marburg Virus Infection

9:20 Travis Warren (DoD/USAMRIID)
Considerations for Development of Small Molecular Nucleoside Analogs for Protection Against Filovirus Disease

9:45 Larry Zeitlin (Mapp Biopharmaceutical, Inc.)
Development of a Monoclonal Antibody Cocktail (MB-003) for Ebola Virus

10:10 Networking break and poster viewing

10:40 Gary Kobinger (Public Health Agency of Canada)
Generating Therapeutic Monoclonal Antibodies Against Ebola Virus

11:05 Lijun Rong (University of Illinois-Chicago)
Discovery of Small Molecule Entry Inhibitors for Filoviral Infections

11:30 John M. Dye (DoD/USAMRIID)
Mammalian and Plant-Derived Antibody-Based Therapies against Sudan Ebolavirus.

12:00 Lunch and poster viewing

Friday, August 23 Afternoon Session

Human disease experiences

Chair, William Dowling (NIH/NIAID/DMID)

1:00 FANG human studies sub-group update, William Dowling (NIH/NIAID/DMID)

1:15 Jens Kuhn (NIH/NIAID)
Filovirus disease outbreaks in numbers – real and perceived threat differences?

1:45 Pierre Rollin (CDC)
Filovirus outbreaks: field challenges

2:15 Panel discussion about the filovirus MCM pipeline

Panel Description/Objectives: Product Development is difficult in the best of circumstances. For Filovirus countermeasure development, with multiple species and variants, requirement for BSL4 containment efficacy studies and demonstration of efficacy using the Animal Rule, product development is particularly challenging. Panelists are asked to address the following general questions and provide their perspectives on how Filovirus MCM development can be facilitated.

Summary Panel Questions:

1. Based on your experience and perspectives, what "lessons learned" did you hear?
2. Based on your experience and perspectives, what gaps/challenges/bottlenecks did you identify?
3. To the extent possible, please make recommended solutions to those gaps/challenges/bottlenecks?
4. Based on your experience and perspectives, what potential next steps would you recommend, either generally or for specific products?

Chair, Ed Nuzum, (NIH/NIAID/DMID)

Panelists

- Annie Frimm (SIGA Technologies)
- Jean Patterson (Texas Biomedical Research Institute)
- David Aglow (BARDA)
- Mary Kate Hart (DynPort Vaccine Company, LLC)
- Tom Ksiazek (University of Texas Medical Branch)

3:30 Adjourn
